# Autoallergy in chronic rhinosinusitis and its clinical relevance^☆^

**DOI:** 10.1016/j.waojou.2026.101382

**Published:** 2026-04-18

**Authors:** Jorge Sánchez, Andres Sánchez, Julian Arango, Dalgys Martinez, Leonardo Puerta

**Affiliations:** a Group of Clinical and Experimental Allergy, Hospital “Alma Mater de Antioquia”, University of Antioquia, Medellín, Colombia; b Group of Medical Investigation (GINUMED), Immunology Department, University Corporation Rafael Núñez, Cartagena, Colombia; c Medical and Experimental Mycology Group, Corporation for Biological Research (CIB). University of Antioquia, Medellín, Colombia; d School of Microbiology, Institution: University of Antioquia, Medellín, Colombia; e Institute for Immunological Research, University of Cartagena, Cartagena, Colombia

**Keywords:** Allergy, Autoimmune, Eosinophils, Immunoglobulin E, Rhinosinusitis

## Abstract

**Background:**

The presence of IgE autoantibodies against human proteins (“autoallergy”) has been identified in different type 2 inflammation.

**Objective:**

The aim of this study was to evaluate the presence of autoallergy in chronic rhinosinusitis (CRS) and explore its clinical impact.

**Methods:**

Cross-sectional study with CRS patients and healthy controls. Three steps were followed: 1) IgE autoantibodies against Fatty Acid Binding Protein (FABP), FABP3 and FABP4, Eosinophil Peroxidase (EPX), and Eosinophil Cationic Protein (ECP) were measured. 2) The allergenic activity of these IgE autoantibodies was evaluated with basophil activation test (BAT) and skin prick test (SPT). 3) Association of these autoantibodies with some clinical outcomes was explored.

**Results:**

22.3% in the CRS group and 12.1% in the control group had at least 1 IgE autoantibody (IgE-AA). In CRS and control group, the most frequent IgE-AA were against EPX (15.9% versus 5.4% *p* = 0.03) and ECP (12.7% versus 4% *p* = 0.04). The IgE autoantibodies had allergenic activity according to BAT and SPT. In CRS group, IgE-AA were associated with higher risk of nasosinusal polyps, clinical severity (SNOT22), asthma, and hyposmia. IgG and IgG4 autoantibodies against these human proteins did not present significant differences between CRS and control group or associations with clinical outcomes.

**Conclusion:**

IgE autoantibodies are presented in CRS patients and are associated with severe clinical outcomes. Accordingly, autoallergy could be an endophenotype in CRS with clinical relevance as a biomarker of disease activity. Further studies with larger cohorts are warranted to validate these findings.

## Graphical abstract

**Figure:**
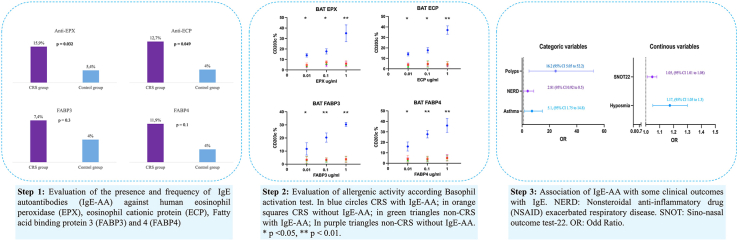

In patients with rhinosinusitis and in a control group, we evaluated the presence of IgE autoantibodies against four proteins (EPX: Eosinophil peroxidase. ECP: Eosinophil cationic protein. FABP3 and FABP4: Fatty acid binding protein 3 and FABP4); we also assessed their allergenic activity and their association with clinical outcomes.

## Introduction

Chronic rhinosinusitis (CRS) is a high impact disease that affects approximately 3–8% of population.^1^^,^^2^ It consists of inflammation in the mucosa of the nose and paranasal sinuses that leads to a set of symptoms with a high individual impact on the quality of life, work activities, and social activities.^3^^,^^4^

In CRS there are different endotypes defined by the underlying inflammation profile, with type 2 inflammation (T2 inflammation) being the most frequent endotype, and it is associated with more severe symptoms such as the coexistence of nasal polyps, severe asthma, and nonsteroidal anti-inflammatory drug (NSAID)-exacerbated respiratory disease (NERD). 5, 6, 7

In T2 inflammation, specific IgE against environmental allergens and recruitment of eosinophils are important mediators of symptoms.^8^^,^^9^ Besides, in the last 2 decades IgE autoreactivity against human proteins (autoantigens) and its role in inflamation have been demostrated in different T2 inflammation diseases.^10^^,^^11^ In atopic dermatitis, IgE autoantibodies are associated with severity, chronicity and it also appears to be a prognostic biomarker of therapeutic response.^12^^,^^13^ In asthma, it was shown that the presence of IgG an recently IgE autoantibodies could be associated with greater severity and greater impairment of lung function.^10^^,^^14^ In CRS, some studies identify IgG against usually targeting autoantigens expressed during the inflammatory response.^15^, ^16^, ^17^ Although the T2 inflammatory response predominates in CRS, To our knowledge, this article is the first to describe IgE autoantibodies in CRS.

During the T2 inflammatory process, an influx of cells especially eosinophils are common in mucosa of CRS patients.^18^^,^^19^ Eosinophils can release inflammatory mediators, such as major basic protein, eosinophil peroxidase (EPX) and eosinophil cationic protein (ECP).^20^^,^^21^ In patients with respiratory T2 inflammation, fatty acid binding proteins (FABPs), has been shown to play an important role in cell migration and release of preformed eosinophil granules in asthma,^22^ additionally, human FABPs and house dust mites FABPs had IgE cross reactivity increasing the probability of “autoallergy”.^23^^,^^24^ Considering that during T2 inflammation there is an increase in the expression of polyclonal immunoglobulin E, and that there is circulation of proteins normally hidden or expressed in low quantities (EPX, ECP, FABPs), it is likely that IgE recognition of these autoantigens occurs, favoring the inflammatory process and its chronicity. Some background in CRS supports this hypothesis; Tsybikov et al described the presence of IgA autoantibodies directed against IL5, which is overexpressed in patients with T2 inflammation;^25^ Schyver et al described that IgG anti-dsDNA autoantibodies in CRS were particularly elevated in populations with high levels of IgE and high levels of EPX.^26^

The aim of this study was to investigate the presence of IgE autoantibodies against FABP3, FABP4, EPX, and ECP in CRS patients, evaluate its allergenic activity and explore their possible association with clinical outcomes.

## Material and methods

### Study design and population

A cross-sectional study was performed using 2 groups: CRS patients recruited in Bogotá and Medellin, Colombia, were enrolled in the RRReNet cohort (Rhinitis and Rhinosinusitis Research Network).^8^ Sampling was done by convenience; all participants in the RRReNet Cohort were invited to donate a blood sample for the present study. CRS groups were patients older than 18 years with primary CRS according to the clinical criteria proposed in the EPOS guidelines^1^ and confirmed by tomography or sinonasal endoscopy. Patients with a medical condition that could affect the interpretation of the clinical scales (for example, congenital or post-trauma nasal cavity anomaly, peripheral neuropathies, primary ciliary atrophy, cystic fibrosis, selective immunodeficiencies) were excluded.

Control subjects, recruited from the same geographic area as the CRS patients and had a similar distribution in terms of age and sex, without documented history of CRS, asthma, or any other chronic respiratory disease was an essential requirement.

All participants were invited to donate a blood sample and undergo a skin prick test (SPT); those who agreed were asked to avoid medications that could potentially interfere with the tests, such as systemic steroids or antihistamines, prior to the SPT. SNOT-22 and ACT were administered an information about medication and sociodemographic characteristics was recollected.

### Determination of IgE, IgG, and IgG4 autoantibodies

FABP3, FABP4, ECP and EPX were obtained as recombinant proteins using an *Escherichia coli* BL21 (DE3) expression system as previously reported.^10^^,^^27^^,^^28^ We explored the presence of IgE autoantibodies by home-made ELISA technique after recombinant protein purification and evaluation of specificity using irrelevant proteins as non-specific binding controls.^11^^,^^27^ The serum used for the quantification of IgE were previously depleted of IgG by immunoaffinity depletion. Residual IgG contents in sera were measured by enzyme-linked immunosorbent assay (ELISA) assays and sera were accepted for IgE-AA measurement when IgG were found to be less than 10 μ/ml In previous studies we have observed that this concentration does not affect the measurement of IgE-AA.^10^^,^^27^^,^^28^ For ELISA, 100 μl of serum was loaded into wells coated with the relevant antigen, and the plates were incubated overnight. Detection was carried out using an alkaline phosphatase-conjugated anti-IgE antibody and *p*-nitrophenol phosphate substrate; the enzymatic reaction was stopped with HCl. The results were expressed in optical density units (OD); The absorbance at 405 nm was determined using a spectrophotometer. Standardization process of this home-made ELISA included 60 healthy atopic and 60 nonatopic subjects and the cut-off value for IgE autoantibodies were defined as the mean plus 3 standard deviations to reduce the risk false positives^11^ (IgE against EPX OD 0.354, ECP OD 0.279, FABP3 OD 0.220, and FABP4 OD 0.224).

For the determination of IgG and IgG4 autoantibodies, the same protocol was followed, except for the IgG depletion; the human sera were previously adsorbed with *E. coli* lysate (diluted 1:50) and the secondary antibody was alkaline phosphatase-conjugated anti-IgG (Pharmigen); in this way only, human IgG was detected. The cut-off values for IgG and IgG4 autoantibodies are presented in supplemental material (Table S1).

### Clinical evaluation

Clinical information of the patients was collected through clinical assessment tools; SNOT22 (Sino-Nasal Outcome Test)^29^ for CRS activity, and VAS (Visual analogue scale) from “0” anosmia to “10” normal for smell assessment;^1^^,^^30^ hyposmia was considered with ≤7 points. In patients with asthma, ACT (Asthma Control test) was measured.

### Evaluation of allergenic activity

Peripheral blood basophils were obtained from some subjects from each group. We used a CD203c expression protocol previously reported.^27^ The percentage of CD203c expression was defined as the percentage of basophils expressing more CD203c than the critical point, which was ≥10.0% of the basophils incubated with buffer only.^27^ Anti-IgE stimulation as a positive control during standardization. Skin prick test (SPT) with FABP3 and FABP4 was proved in concentration of 25 ng/ul of each protein, 50% glycerol, and 0.4% phenol. This protein concentration was chosen after dose titration with different concentrations was done in 10 patients and 10 controls.^28^ We conducted a pilot test on 20 healthy subjects to evaluate the feasibility of performing SPT with EPX and ECP; however, we observed a local irritative effect in 4 of the 20 patients attributable to their protease activity, so we decided not to perform the evaluation on the rest of the patients.

### Bioethical considerations

The study was approved by the Institutional Ethics Committee (code IN48-2021, act #179, Hospital “Alma Mater de Antioquia”) and conducted in accordance with the Declaration of Helsinki. Written informed consent was obtained from all participants.

### Statistical analysis

For sample size calculation, there are no previous articles on CRS that allow for estimating a probable prevalence. Based in previous studies on other type 2 diseases as reported in Sanchez et al, Mukherhee et al, and Chan et al, 10, 31, 32 the prevalence of IgE-AA in CSR could be between 5% and 30% (median 10%) and in general population of 0–10%; considering the principal aim of this study ∗(demonstrate the presence of IgE autoantibodies in CRS patients) a sample size of 80 patients will allow the identification of at least 8 patients (4–24) with IgE-AA in CRS, which would allow for evaluation of allergenic activity and to explored autoantibodies relationship with some clinical characteristics.

Absolute and relative frequencies, and the median or the interquartile range, were used for the descriptive analysis. For categorical variables the Pearson chi-square test of independence was applied or Fisher's exact test when was appropriated. Mann-Whitney *U* test and Kruskal–Wallis test was used for comparisons between continuous variables. For comparisons of continuous variables with small sample size Mann-Whitney-Wilcoxon tests or t de Student according to the distribution. Correlations were assessed with Spearman coefficient (r). A *p* value ≤ 0.05 was considered statistically significant. Statistical analyses were performed using IBM SPSS Statistics for Windows, version 26.0 (IBM, Inc, Chicago, IL), JAMOVI 2.3.38 (Sydney, Australia) and GraphPad Prism 9 (La Jolla, CA). Fo evaluation of clinical outcomes association with autoantibodies, a proof-of-concept evaluation (PoE) was done; PoE is a small-scale experiment or demonstration to explore the viability and potential of an idea, method, or product before investing significant resources in its full development.

## Results

### General characteristics

A total of 94 CRS patients and 74 controls participated (Table 1). CRS group had a predominance of female sex (59%), and the median age was 42 years. This group had higher levels of total IgE (mean 162 vs 108 IU/ml, *p* < 0.001) and blood eosinophils (mean 233 vs 134 cells/ml *p* < 0.001) than the control group. IgE sensitization to environmental allergens (Table 1) was present in 62.7% and 21.6% of the CSR group and of control group respectively (p < 0.001). Hyposmia was the most frequent comorbidity (56.3%) followed by asthma (31%), history of current or past polyposis (23%), and NERD (19%) (Table 1). The median of SNOT22 was 25 points. Among patients with asthma ACT median was 19 points.

**Table 1:** **General characteristics**.

		CRS (n = 94)	Non-CRS (n = 74)	*p*
CRS duration	*Months*	Me 18 (IR 32)	No apply	No apply
Sex	*Female*	55 (59%)	43 (58%)	0.9
	*Male*	39 (41%)	31 (42%)	
Age group		Me: 42 (IR: 14)	Me: 41 (IR:17)	
Comorbidities	*Asthma*	39 (41%)	NA	NA
	*NERD*	18 (19%)	NA	NA
	*Polyps (past or present)*	22 (23%)	NA	NA
Symptoms	*SNOT22*	Me: 25.5 (IR: 18.5)	NA	NA
	*Hyposmia (VAS)*	Me: 5 (IR: 5)	NA	NA
	*ACT*	Me: 19 (IR: 5)	NA	NA
Atopy		59 (62.7%)	16 (21.6%)	<0.001
Biomarkers	*Total IgE*	162 (IR: 71)	Me: 108 (IR: 71)	<0.001
	*Blood eosinophils*	233 (IR: 88)	134 (IR: 73)	<0.001
	*sIgE Der p*	51 (54.2%)	11 (14.8%)	<0.001
	*sIgE Der f*	50 (53.1%)	11 (14.8%)	<0.001
	*sIgE Blo t*	23 (24.4%)	9 (12.1%)	0.04
	*sIgE Dog*	14 (14.8%)	1 (1.3%)	0.002
	*sIgE Cat*	12 (12.7%)	2 (2.7%)	0.01
	*sIgE cockroach*	10 (10.6%)	3 (4%)	0.1
	*sIgE grasses*	8 (8.5%)	0	0.01
	*Enterotoxin A*	10 (10.6%)	0	0.002
	*Enterotoxin B*	10 (10.6%)	0	0.002

Sociodemographic characteristics of CRS group and control group. Atopy was defined as the presence of sIgE in serum for at least 1 allergen (*Blomia tropicalis*, *Dermatophagoides pteronyssinus*, *Dermatophagoides farinae*, Dog, Cat, Cockroach, grasses, Enterotoxin A, or enterotoxin B). NA: no apply. sIgE: specific IgE in serum. IR: Interquartile range

### Presence of IgE autoantibodies

**Fig. 1:**
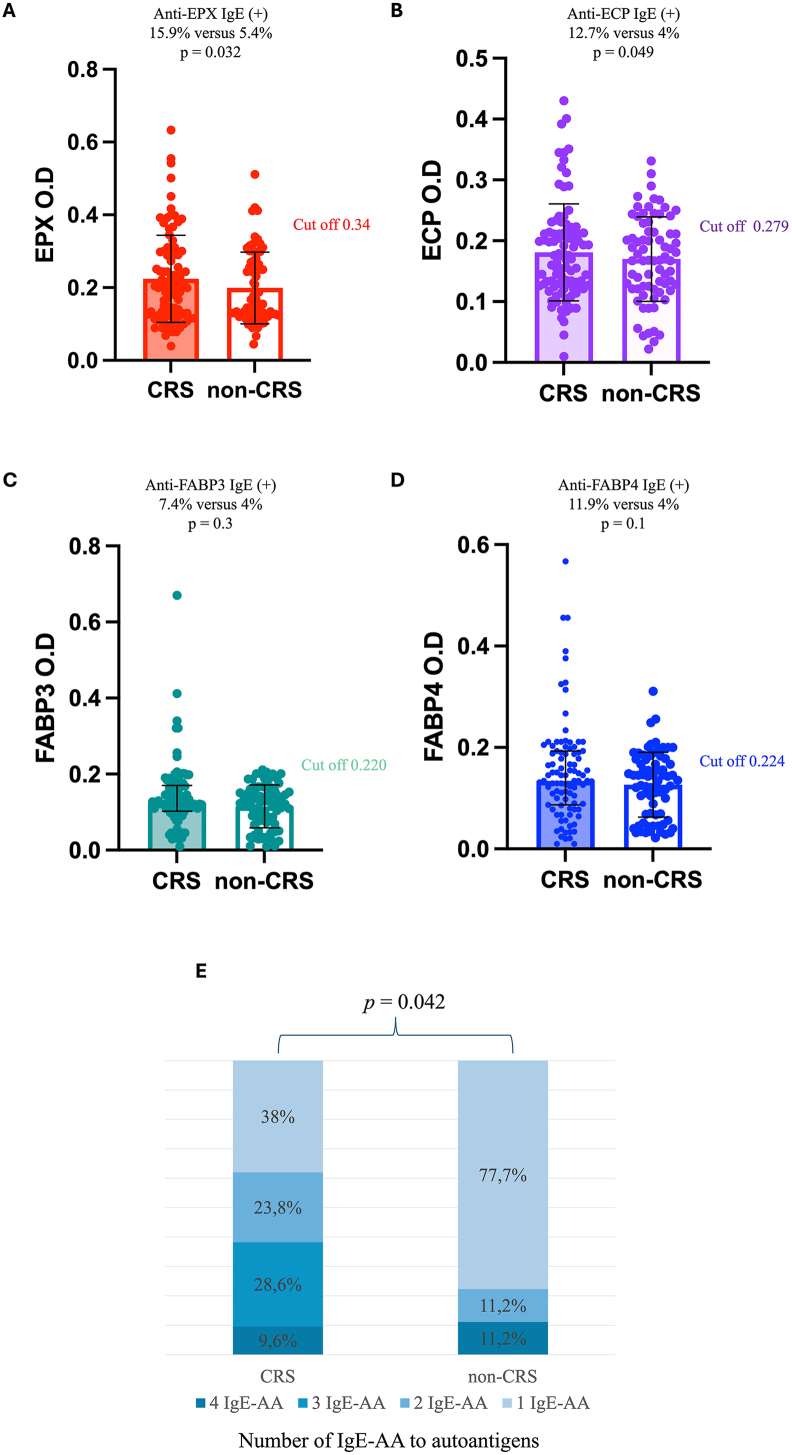
**IgE autoantibodies against EPX, ECP, FABP3, FABP4**. Concentration of IgE-AA (IgE autoantibodies) according to Optical Density (O.D). The percent represents patients with positive Ig-AA in CRS group and non-CRS. Statistically significant were calculated according to the frequency of each IgE-AA in each group. In figure E, the colored box indicates the percentage of patients with sensitization to 1, 2, 3, or all 4 autoantigens: “1 IgE-AA”: One positive autoantibody; “2 IgE-AA”: 2 positive autoantibodies; “3 IgE-AA”: 3 positive autoantibodies; “4 IgE-AA”: Four positive autoantibodies. ∗*p* significance >0.05.

Twenty-one CRS patients (22.3%) and 9 (12.1%) control subjects had positive IgE-AA to at least 1 of the recombinant proteins. Serum IgE-AA levels to EPX and ECP were significantly higher in CRS group than those in control group (Fig. 1). IgE-AA sensitization to more than 1 autoantigen was more frequent in the CRS group (*p* = 0.042) (Fig. 1). In CRS group, IgE-AA serum levels against EPX and ECP presented a low correlation (R 0.350 *p* < 0.001). Patients with IgE-AA against FABPs had a higher frequency of sensitization to house dust mites (HDM) (84%) than patients with IgE-AA against ECP or EPX (73%) but it was not statically significant.

### IgG against eosinophil autoantigens

**Fig. 2:**
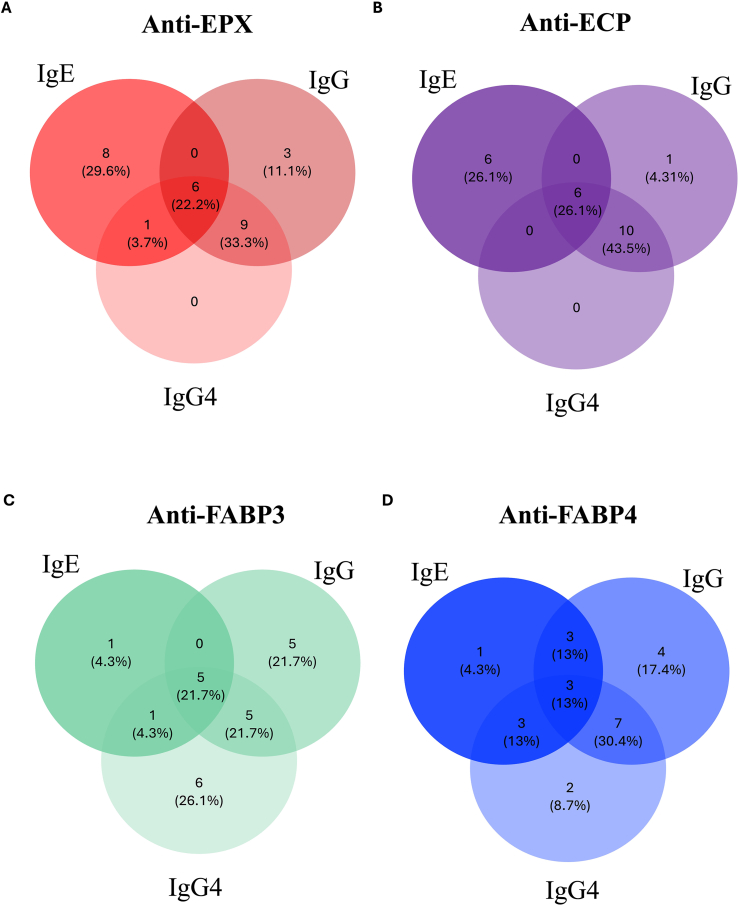
**Interactions of IgE, IgG and IgG4 autoantibodies**. Venn Diagram showing distribution of patients with IgE, IgG and IgG4 autoantibodies (AA), against EPX, ECP, FABP3, and FABP4.

Frequency of positive IgG and IgG4 autoantibodies (AA) are presented in the supplemental material (Table S1). IgG and IgG4 autoantibodies did not present significant differences between CRS and control group or associations with clinical outcomes (Table S2). In CRS patients IgG4-AA against EPX or ECP was always accompanied of IgE or IgG reactivity against these proteins. CRS patients had IgG4-AA against FABP3 (26.1%) and against FABP4 (8.7%) (Fig. 2). The 9 control subjects who had IgE-AA, also had IgG4-AA (2–3 times higher) when compared with the rest of the controls, however, the number of patients was not sufficient to evaluate statistical significance (Data not shown).

### Allergenic activity

**Fig. 3:**
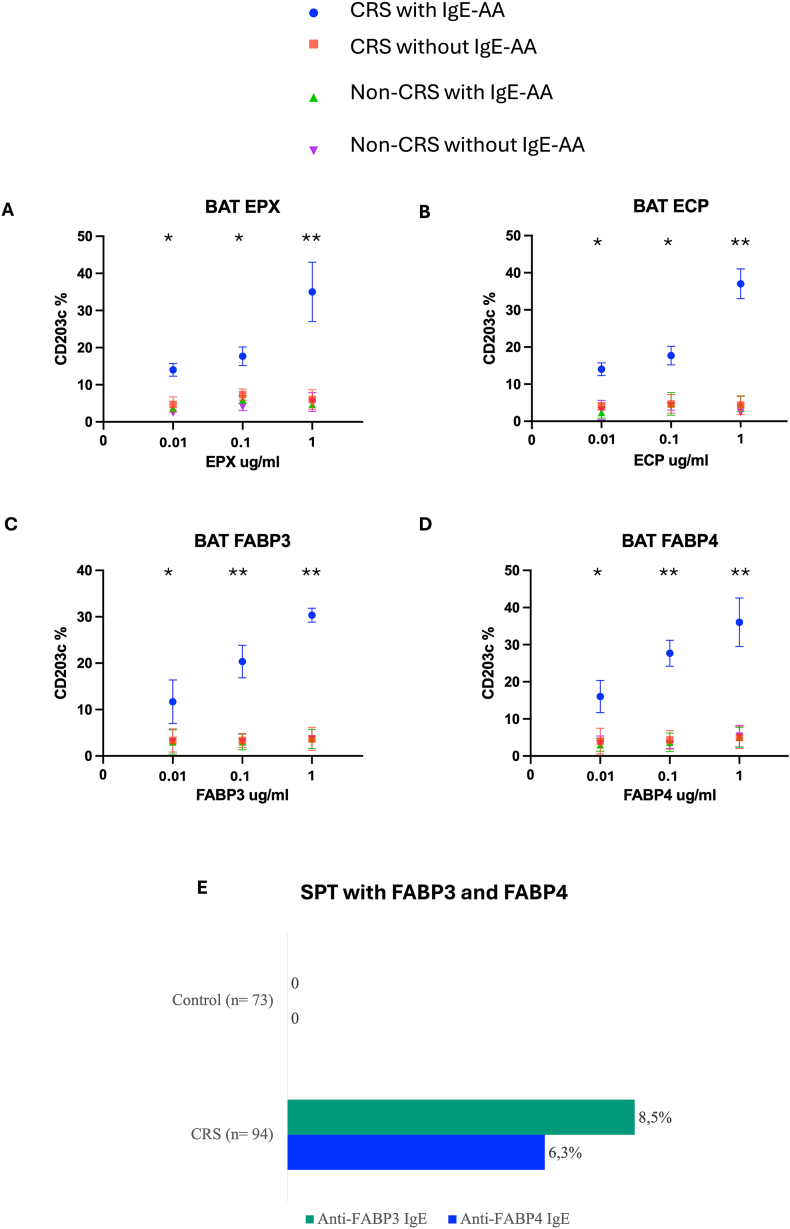
**Allergenic activity according to BAT and SPT**. Allergenic activity according basophil activation test (BAT) was evaluated according to CD203 expression using 4 autoantigens (EPX, ECP, FABP3, FABP4). Only the Chronic rhinosinusitis (CRS) group with IgE-AA presented BAT Activation. In figure E, frequency of positive skin prick test (SPT) between control group and CRS group for FABP3 and FABP4. ∗*p* < 0.05, ∗∗*p* < 0.01.

For basophils activation, 4 groups were defined: CRS with IgE-AA (n = 4), CRS without IgE-AA (n = 4), non-CRS with IgE-AA (n = 4), and non-CRS without IgE-AA (n = 4) (Fig. 3). Each AA induced CD203 expression in the CRS with IgE-AA group. In contrast, none of AA induced basophil degranulation in samples from CRS without IgE-AA, non-CRS with IgE-AA, and non-CRS without IgE-AA.

SPT using FABPs was done in 73 control subjects and 94 CRS (Fig. 3). Eight (8.5%) and 6 (6.3%) CRS patients showed a positive SPT to FABP3 and FABP4 respectively; all of them had blood ant-FABP3 or FABP4. None of the control subjects showed a positive SPT even those subjects with positive anti-FABP3 IgE or anti-FABP4 IgE (Fig. 3).

### Clinical relevance of autoantibodies

As an exploratory proof-of-concept evaluation, the potential association between IgE autoantibodies (IgE-AA) and selected clinical variables was assessed in the CRS group.

For this purposes, clinical outcomes with a *p* < 0.1 were included in the risk analysis evaluation. Considering that most CRS patients with IgE-AA were sensitized to more than 1 autoantigen, the 4 autoantigens were evaluated as a group but when evaluated each IgE-AA separately or by groups (FABPs and Eosinophil IgE-AA) association persisted for ECP, EPX and FABPs when were evaluated together.

**Fig. 4:**
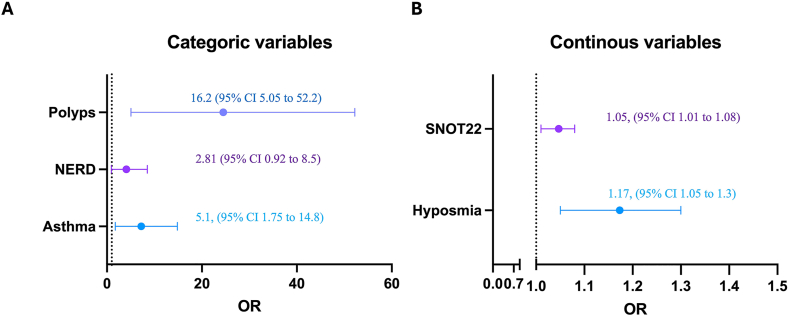
**IgE-AA and clinical outcomes**. The Odd Ratio (OR) of some clinical outcomes was associated with the presence of IgE autoantibodies (IgE-AA). NERD: Nonsteroidal anti-inflammatory drug (NSAID) exacerbated respiratory disease. SNOT: Sino-nasal outcome test-22.

IgE-AA was associated with different comorbidities (Table 2). CRS patients with IgE-AA had a higher risk for asthma (OR 5.1 95% CI 1.75 to 14.81, *p* = 0.002), and nasal polyps (OR 16.2 95% CI 5.05 to 52.2, *p* < 0.001) (Fig. 4) after adjusting for confounding factors trough multivariate analysis.

**Table 2:** **Characteristics of CRS patients according to IgE-AA**.

		CRS IgE-AA (+) (n = 21)	CRS IgE-AA (−) (n = 73)	*p*
CRS duration	*Months*	Me 22 (IR 32)	Me 17 (IR 28)	0.14
Sex	*Female*	12 (57.1%)	43 (58.9%)	0.88
	*Male*	9 (42.8%)	30 (41%)	
Age (years)		Me: 38 (IR: 11)	Me: 44 (IR: 17)	0.08
Comorbidities	*Asthma*	15 (71.4%)	24 (32.8%)	0.002
	*NERD*	7 (33.3%)	11 (15%)	0.06
	*Polyps (past or present)*	14 (66.6%)	8 (10.9%)	<0.001
Symptoms	*SNOT22*	Me: 43 (IR: 17)	Me: 23 (IR: 16)	<0.001
	*Hyposmia (VAS)*	Me: 3 (IR: 4)	Me: 6 (IR: 4)	0.03
	*ACT*	Me: 19 (IR: 4)	Me: 20 (IR: 6.5)	0.6
Atopy		16 (76.1%)	43 (58.9%)	0.14
Biomarkers	*Total IgE*	127 (IR: 116)	128 (IR: 126)	0.9
	*Blood eosinophils*	224 (IR: 135)	224 (IR: 88)	0.5

Sociodemographic and clinical characteristics of chronic rhinosinusitis (CRS) patients according to positive or negative IgE-AA (IgE autoantibodies). Atopy was defined as the presence of IgE in serum for at least 1 environmental allergen. sIgE: specific IgE in serum. IR: Interquartile range

Additionally, no significant association was found between NERD and IgE autoantibodies (IgE-AA) (OR 2.81, 95% CI 0.92 to 8.5, p = 0.06). Significant associations were observed between the presence of IgE-AA and both the SNOT-22 scores (p < 0.001) and VAS scores for hyposmia (p < 0.03). As these variables are continuous, the risk of having IgE-AA increases with each additional point on the respective scales. When compared CRS patients with and without IgE-AA there was not association with the total IgE levels or blood eosinophil counts (Table 2).

## Discussion

In CRS the role of IgE has been studied as part of T2 inflammation.^33^, ^34^, ^35^ Sanchez J et al observed with nasal challenge test that sIgE against HDM is clinically relevant in CRS patients, suggesting that sIgE has a relevant role in some patients.^8^ In other T2 inflammatory disease like asthma, sIgE-AA have been identified and associated with low remission rate and severe presentations.^10^ Nevertheless, to our knowledge, this is the first study evaluating the presence of sIgE AA in CRS and their potential clinical utility. Previous studies suggest that FABPs, EPX, and ECP, are associated with type 2 inflammatory response:^10^^,^^11^^,^^22^^,^^28^ FABPs are intracellular proteins that bind to fatty acids participating in their transport and regulation.^36^^,^^37^ Demir et al found that serum FABP4 levels may reflects neutrophilic asthma.^38^ During an inflammatory process, FABP3 and FABP4 could be exposed in the extracellular space and be recognized by IgE.^10^^,^^11^^,^^22^^,^^28^ In this study we demonstrated that anti-FABP3 IgE and anti-FABP4 IgE had allergenic capacity in vitro test (BAT) and in vivo (SPT) suggesting that these autoantibodies are associated with inflammatory response in CRS. Human FABPs have homologs in allergen group 13 of HDM; Smole U et al observed that Blo t 13, a *Blomia tropicalis* FABP, could boost the T2 response at the level of the respiratory tract by binding to the serum amyloid A1 (SAA1) receptor.^39^ Collectively, these results suggest that IgE-AA against FABPs could be the result of cross-reactivity between FABPs from human and HDM species. Additionally, most of CRS with IgE-AA had also HDM IgE sensitization, supporting the possibility that HDM sensitization and cross-reactivity may be and important mechanism for the development of autoantibodies to FABPs, similar to the “molecular mimicry” described in some autoimmune diseases.^40^ IgG4 antibodies are usually the result of prolonged antigen recognition. Some patients had IgG4-AA against FABP3 and FABP4 without anti-FABPs IgE. Suggesting additional mechanisms for formation of these autoantibodies.^40^

Why IgE-AA are formed is unknown but for EPX and ECP cross-reactivity with environmental allergens seems unlikely and other mechanisms could explain the development of autoantibodies against EPX and ECP for example “autoantigen overproduction”;^40^^,^^41^ EPX and ECP are eosinophil intracellular proteins that are expressed during T1 and T2 inflammation and are released by eosinophils into the extracellular space in tissues when inflammatory process occurs.^42^^,^^43^ Our hypothesis is that during T2 inflammation occurs extracellular overexpression of these proteins normally hidden at intracellular level and due to the IgE clonal diversity stimulated by T2 inflammation, IgE have a greater chance of recognizing these human proteins and increasing their affinity against them.^10^ This hypothesis is supported by previous studies in asthma, atopic dermatitis, and urticaria, where IgE autoantibodies have been detected against some eosinophil-related proteins usually expressed in low levels in healthy people.^10^^,^^11^^,^^44^ Besides, autoantibodies against EPX and ECP may impair the proper functioning of these proteins and, consequently, might promote the formation of immune complexes. This mechanism might exacerbate tissue damage, resembling the pathological processes observed in autoimmune diseases characterized by type III hypersensitivity.^45^^,^^46^ Nevertheless, the current study identifies associations between IgE-AAs and disease severity, but cannot determine whether IgE-AAs are drivers of disease progression or secondary to ongoing inflammation. The lack of association between blood eosinophil levels and the presence of IgE autoantibodies indicates that this mechanism is not dependent on eosinophil concentration, so other factors such as the type of tissue damage, the duration of inflammation, and the activity of inflammation may play a part in this endotype.

Our results suggest that IgE-AA are related with the severity of CRS. We observed that all of them had allergenic capacity according to basophil activation test and anti-FABPs IgE can also induce hives during SPT indicating histamine release. Additionally, IgE IgE-AA were associated with various clinical outcomes related to CRS. Although the association with clinical outcomes was exploratory and requires further validation using a larger number of cases and controls, our results suggest that IgE-AA could serve as predictive biomarkers of CRS severity, with potential utility in clinical practice. The lack of association between NERD and the presence of IgE-AA appears to be secondary to the low number of subjects available for the comparison analysis; despite this, we observed a trend (2.81 95% CI 0.92 to 8.5, p 0.06) which for a proof-of-concept analysis indicates a possible association but it should be confirmed in future studies.

IgG-AA are usually more abundant that IgE-AA in general population.^47^ There is little information about the role of IgG-AA in CRS^16^^,^^48^ but increasing evidence suggests that autoimmune disorders may increase the risk of rhinosinusitis.^49^^,^^50^ According to our results, IgE autoantibodies, but not IgG/IgG4, are clinically significant in CRS. This could be explained by the fact that rhinosinusitis, like asthma and rhinitis, shares a predominantly T2 inflammatory profile, favoring an IgE reactive response over other antibodies. Furthermore, it is important to note that this lack of IgG-AA association may be limited to the antigens observed in this study but could be relevant to others. Asamori et al suggest that the pathobiology of CRSwNP involves autoreactive humoral immunity and may be molecular mimicry could drive these autoimmune response.^51^ However, the identification in CRS patients of IgG autoantibodies against proteins such as IL5 and anti-phospholipid suggests that other mechanisms could be involved in this autoreactive response.^17^^,^^25^ IgG-AA has been detected in other T2 diseases but it's clinical relevance its controversial because it is not always associated with clinical outcomes.^52^ Similar to a previous report in ,^11^ we detected IgG-AA in CRS and were in higher levels than IgE-AA but there were not associated with CRS or with clinical outcome. An interesting finding that needs to be confirmed in further studies was that among control subjects who had IgE-AA, the levels of IgG4 autoantibodies were high, suggesting a blocking mechanism that would explain the lack of basophil activation in control subjects during BAT. The role of IgG, especially IgG4, in allergies is currently being reconsidered. For example, in eosinophilic esophagitis, some studies suggest it may play a role in the inflammation of the disease, and in asthma, its relationship as a possible autoantibody has been evaluated.^52^^,^^53^ However, the most robust evidence in T2 inflammation indicates its possible role as a counterregulatory antibody, competing for recognized antigens with IgE but also forming low-affinity binding sites on membrane receptors.

Our study has some limitations and strengths. This is the first study evaluating IgE-AA in CRS and its allergenic activity. Although we were unable to perform a standardization to evaluate EPX and ECP proteins through skin tests, we used the basophil activation test. The studies conducted regarding the association of autoantibodies with certain clinical outcomes are useful as a starting point, nonetheless we recognized that further studies need to be done to assess their clinical usefulness. Considering that multiple clinical comparisons were done, a false-positive risk should be considered. However, this exploration is a proof-of-concept evaluation; and can be used as a starting point for subsequent analysis. Additionally, despite clinical outcomes associated with IgE-AA (Polyps, NERD, Asthma, SNOT22, Hyposmia) are closely interrelated, association persist after adjusted in multivariable analysis. The study focused on only 4 autoantigens (FABP3, FABP4, EPX, and ECP), which may not fully represent the complexity of IgE-mediated autoimmunity in CRS. The possibility of additional, clinically relevant autoantigens warrants further investigation.

Despite these limitations, this study provides novel insights that open new avenues for research aimed at better understanding the pathogenesis of CRS, as well as identifying potential diagnostic, prognostic, and therapeutic targets. Additionally, most studies evaluating the presence of autoantibodies have been performed on nasal polyp samples; our study included patients with and without polyps, which strengthens the assessment of the clinical utility of IgE autoantibodies and measuring them in blood could facilitate access to this test in the future.

In conclusion, IgE-AA seems to play an important role in the pathogenesis of CRS and are associated with severity. These results deserve to conduct investigations to confirm the clinical implications in CRS.

## Abbreviations

ACT: Asthma control test; BAT: Basophil activation test; Blo t: *Blomia tropicalis*; CRS: Chronic rhinosinusitis; Der p: Dermatophagoides pteronyssinus; Der f: Dermatophagoides farinae; EPX: Eosinophil peroxidase; ECP: Eosinophil cationic protein; FABP3: Fatty acid binding protein 3; FABP4: Fatty acid binding protein 4; HDM: House dust mites; IgE: Immunoglobulin E; IgE-AA: Immunoglobulin E autoantibodies; IgG-AA: Immunoglobulin G autoantibodies; NERD: Nonsteroidal anti-inflammatory drug (NSAID)-exacerbated respiratory disease; NSAID: nonsteroidal anti-inflammatory drug; SPT: Skin prick test; SNOT22: Sino-nasal outcome test (SNOT-22); sIgE: specific Immunoglobulin E; VAS: Visual analog scale.

## Authors contributions

Jorge Sanchez contributed the central idea. All authors contributed equally to the execution and writing of the manuscript.

## Ethical considerations

The Ethics committees of the University of Antioquia and the Clinic “IPS Universitaria”, approved the study (code IN48-2021, act #179, Hospital “Alma Mater de Antioquia”). Written informed consent was obtained from all the participants.

## Data availability

The datasets generated during the current study are available from the corresponding author on reasonable request. The data that support the findings of this study are not publicly available due to recommendation of the ethics committee. Requests for access must be directed to the corresponding author.

## Artificial intelligence disclosure statement

Nothing to disclose.

## Funding

University of Antioquia and “Asociación Colombiana de Alergia Asma e Inmunología” funded the study.

## Conflict of interest

The authors have no relevant financial or non-financial interests to disclose.
