# Supplementary material for: Autoallergy in chronic rhinosinusitis and its clinical relevance
Source: World Allergy Organ J. 2026 Apr 18;19(5):101382. doi: 10.1016/j.waojou.2026.101382 (PMC13098401; doi:10.1016/j.waojou.2026.101382)
Supplement: Multimedia component 1 [file mmc1.docx]

**SUPLEMENTAL MATERIAL**

**Table S1. Comparison of IgG-AA and IgG4-AA between CRS group and control group**

| IgG autoantibodies | Cut-off | CRS group (n 94) | Control group (n 74) | *p^1^* | *p^2^* |
| --- | --- | --- | --- | --- | --- |
| *Anti-EPX IgG* | 0.425 | n 18, 19.1% (Me 0.315 IR 0.129) | n 10, 13.5% (Me 0.222 IR 0.142) | 0.3 | 0.3 |
| *Anti-ECP IgG* | 0.414 | n 17, 18% (Me 0.299 IR 156) | n 9, 12.1% (Me 0.187 IR 0.144) | 0.2 | 0.4 |
| *Anti-FABP3 IgG* | 0.821 | n 15, 15.9% (Me 0.793 IR 0.541) | n 9, 12.1% (Me 0.849 IR 0.649) | 0.4 | 0.6 |
| *Anti-FABP4 IgG* | 0.633 | n 17, 18% (Me 0.754 IR 0.613) | n 9, 12.1% (Me 0.701 IR 0.578) | 0.2 | 0.7 |
| *Anti-EPX IgG4* | 0.189 | n 17, 18% (Me 0.114 IR 0.102) | n 5, 6.7% (Me 0.154 IR 0.084) | 0.068 | 0.7 |
| *Anti-ECP IgG4* | 0.149 | n 17, 18% (Me 0.109 IR 0.070) | n 6, 8.1% (Me 0.102 IR 0.056) | 0.062 | 0.8 |
| *Anti-FABP3 IgG4* | 0.351 | n 13, 13.8% (Me 0.222 IR 0.389) | n 5, 6.7% (Me 0.281 IR 0.421) | 0.1 | 0.6 |
| *Anti-FABP4 IgG4* | 0.273 | n 15, 15.9% (Me 0.233 IR 0.150) | n 7, 9.4% (Me 0.251 IR 0.129) | 0.2 | 0.7 |

**Table S1:** IgG-AA and IgG4-AA levels are presented as optical density. The “*p*^1^” value was based in the frequency of positive autoantibodies between CRS group and control group. The “*p*^2^” value was based in the concentration of autoantibodies between CRS group and control group. Me: Median. IR: Interquartile range.

**Table S2. IgG-AA and IgG4-AA according to IgE-AA.**

| IgG-AAs | Cut-off | CRS IgE-AA (+), n 21 (Me, IR) | CRS IgE-AA (-), n 73 (Me, IR) | *p^1^* | *p^2^* |
| --- | --- | --- | --- | --- | --- |
| *Anti-EPX IgG* | 0.425 | n 6, 28.5% (0.315, 0.109) | n 12, 16.4% (0.352, 0.123) | 0.2 | 0.5 |
| *Anti-ECP IgG* | 0.414 | n 6, 28.5% (0.319, 121) | n 11, 15% (0.287, 0.104) | 0.1 | 0.5 |
| *Anti-FABP3 IgG* | 0.821 | n 6, 28.5% (0.765, 0.342) | n 9, 12.3% (0.801, 0.496) | 0.07 | 0.8 |
| *Anti-FABP4 IgG* | 0.633 | n 6, 28.5% (0.734, 0.333) | n 11, 15% (0.771, 0.482) | 0.1 | 0.8 |
| *Anti-EPX IgG4* | 0.189 | n 5, 23,8% (0.124, 0.112) | n 12, 16.4% (0.101, 0.064) | 0.4 | 0.8 |
| *Anti-ECP IgG4* | 0.149 | n 6, 28.5% (0.103, 0.067) | n 11, 15% (0.112, 0.065) | 0.1 | 0.9 |
| *Anti-FABP3 IgG4* | 0.351 | n 5, 23,8% (0.200, 0.369) | n 8, 10.9% (0.291, 0.391) | 0.1 | 0.8 |
| *Anti-FABP4 IgG4* | 0.273 | n 5, 23.8% (0.253, 0.170) | n 10, 13.6% (0.241, 0.109) | 0.2 | 0.8 |

**Table S2:** CRS patients according to positive or negative IgE-AA. The “*p*^1^” value was based in the frequency of positive autoantibodies between CRS group and control group. The “*p*^2^” value was based in the concentration of autoantibodies between CRS group and control group. Me: Median. IR: Interquartile range.
